# Remarks on “ *Two new species of Tornidae (Caenogastropoda, Rissooidea) from Espírito Santo, Brazil*,” by Luiz Ricardo Simone ( *ZooKeys* 238: 77–85, 2012) and a plea for improvement in ZooKeys editorial policy

**DOI:** 10.3897/zookeys.255.4548

**Published:** 2012-12-28

**Authors:** Richard E Petit

**Affiliations:** 1 806 Saint Charles Road North Myrtle Beach, SC 29582

With comments provided by Terry Erwin, Eike Neubert and Lyubomir Penev

The purpose of this letter is to point out some shortcomings in the editing of a recent *ZooKeys* paper and to discuss broader issues relating to the editorial procedures used by this important journal. The paper of concern, titled “Two new species of Tornidae (Caenogastropodas, Rissooidea) from Espírito Santo, Brazil,” was published by Luiz Ricardo L. Simone in *ZooKeys* 238: 77–85 on 6 November 2012. Grammatical errors begin to appear in the first sentence of the abstract. Not only are sentences sometimes incomplete and improperly constructed, but there are some instances where their meaning cannot be determined. For example, what sense can be made of “since specimens with periostracum to eroded shells” (p. 78)? The language problem is most troubling in the descriptions of the shells where there are constructions such as “On aperture, region between ridge of superior carina and insertion of outer lip in adjacent preceding whorl a small region with ridge of peripheral ridge reabsorbed, forming anal notch with ~ 1/5 of aperture size” (p. 79). Simone is no newcomer to English language publication (see here^1^ for a list of his publications), but it appears that he used a machine translator for portions of this paper instead of relying on an English-speaking colleague.

**Comment:** *ZooKeys has well-defined policies for English language editing. During the submission process*, *authors are warned that manuscripts should be submitted only after being edited by a native English speaker. Authors have to confirm by checking a tick box that they have followed the above requirement. Unfortunately, it happens that some authors provide incorrect information on the language editing of their manuscripts*.

*Involving outsourced language editing services by Pensoft would visibly increase the price of the open access fees charged by the journal, which shall become an additional obstacle for persons and institutions to publish in ZooKeys. Therefore we rely both on the conscience of our authors to provide stylistically proven texts and our editors to filter out badly written manuscripts*.

The first two sentences of the *ZooKeys* Author Guidelines are: “All papers should be in grammatically correct English. Non-native English speaking authors are required to have their manuscripts checked by a native English speaker prior to submission.” Surprisingly this is left to the author as the Guidelines state that “reviewers are not expected to provide a thorough linguistic editing or copyediting of a manuscript.” Removing that burden from reviewers is understandable, but the same wording appears in the instructions for Subject Editors.

**Comment:** *ZooKeys provides basic copy-editing but not linguistic editing of the manuscript. We do not expect that our reviewers and editors should spend their precious time in thorough editing of the English language. Nevertheless, many of them do this on voluntary basis and we greatly appreciate their efforts!*

An unusual periostracum is an important feature of the *Cyclostremiscus* species described by Simone. However, it is not possible to determine exactly what is perisotracum in Figures 1–5 as there is no visible demarcation, or any indication of such, between shell and periostracum. The poor quality of this plate is compounded by the unintelligible description of the species which makes it difficult to understand why all of the shells in Figures 1–15 are treated as conspecific. In the Guidelines, *ZooKeys* lists six items under Focus & Scope. The second of these requires, for a new species, a thorough description with good quality images, neither of which is present in this paper, and the third requires a differential diagnosis. One may assume that Simone performed these tasks in an original Portuguese version of the manuscript, but it should have been properly translated. A fourth item required is an identification key. Keys are rarely used by malacologists and this requirement was obviously, and appropriately, waived for this paper.

**Comment**: *In his text, Simone describes the periostracum in a separate paragraph, and this structure can be clearly seen on the figures cited. On the intermediate carina, these structures are obviously somewhat darker, which might be due to sand grains that adhere to the periostracal rods. He could probably have added an arrow to explicitly pinpoint these structures, however, the description of this peculiar feature was clear to me on the first sight, so there was no direct need to add something. I agree, in the caption this could have been mentioned again. However, I do not agree that this is a plate of poor quality, particularly if you consider the small size of the objects! We all know that it is quite difficult to take pictures of shells with a size of 2.5 mm*.

*Secondly, a differential diagnosis is present. Simone compares his new species to three other species, namely* C. beauii, C. pentagonus *and* C. trilix,* in the paragraph discussion*.

The fifth required item under Focus & Scope of the Authors’ Guidelines is Etymology. Simone describes two new species in his paper. A complete etymology is given for one, but there is no etymology for the new species *Cyclostremiscus mohicanus*. Given the current state of knowledge of the Tornidae it is highly possible that this species may eventually be placed in a different genus. As *mohicanus* is not a Latin word, in the absence of its etymology it may present problems should it be placed in a feminine or neuter genus. The only Brazilian usage of this name located is for its use to describe the haircut style of a noted Brazilian football (= soccer) player. There can be no objection if that was the author’s intent, but is it an adjective (he had a mohican haircut) or a noun (he had a mohican)? How it would be used in Portuguese is not known. I encourage Simone to publish a note providing the etymology of *mohicanus*.

**Comment:** *As explained to me by Dr Simone, the species epithet “mohicanus” is derived from the Indian tribe name “Mohican” and used here as a simple adjective. As the gender of the species epitheton is determined by the gender of the genus, the grammatical form “mohicanus” is correct, because Cyclostremiscus is of male gender. In case this species is transferred to another genus with a female or neuter gender, it turns to “mohicana” or “mohicanum”. In case this name would have been a noun in apposition, it would remain in its original form. This problem could have been avoided by publishing an etymology of the species epithet, and I am grateful to Mr. Petit for drawing our attention to this failure, for which I take responsibility.”*

Every author has had a paper appear in print with typographical errors that should have been caught by him/her and were overlooked by reviewers and editors. Simone’s paper did not escape such error as *Episcynia* is misspelled as *Episcinia* in the abstract and twice on page 81. Also, “(Bush, 1885)” appears in the text as “(Bursch, 1885).” Neither this Bush paper nor a number of other cited papers are listed in the References. An omission of a listing in References of all cited items for a *ZooKeys* paper is strange as the instructions to authors stress the importance of cross-checking all entries “because all references will be linked electronically as completely as possible to the papers cited.” Failure to follow the guidelines, especially in this instance, reflects unfavorably on both the author and the editor and unnecessarily raises the question of whether or not the author ever actually saw a cited work.

The only two other papers in *ZooKeys* that are on non-opisthobranch shelled marine gastropods (Caballer et al. 2011; Dornellas 2012) also show editing lapses. In the paper by Caballer et al. (2011) the abstract is difficult to read because several sentences begin with abbreviations. Two sentences (p. 1), in part, are: “… *Rissoella venezolanicola* sp.n.*R. morrocoyensis* sp. n. …”. The “*R*.” actually begins a sentence. This may be journal policy, but if so the propriety of changing that policy should be examined. The practice of beginning a sentence with a nonacronymic abbreviation is considered improper in the CBE Style Manual and in all English grammars.

The technically excellent paper by Dornellas (2012) is marred by the citation of Swanson instead of Swainson in the Introduction. The misspelled word radichian (instead of rachidian) would possibly escape notice were it not in bold face type. Quinn’s *Calliostoma axelolssoni* is unfortunately corrupted to *C. axelsonni*. Again, in this paper there are many citations of papers that are not listed in the References.

**Comment:** *As stated above, ZooKeys Editorial Office provides basic copy-editing for each manuscript, during which many errors and inconsistencies are being corrected. It may happen that some of the errors are overlooked, mostly due to the increase of the amount of work with the journal’s exponential growth (see for detail Erwin et al. 2011, Erwin et al. 2012)*.

*Implementing of thorough copy-editing services would visibly increase the open access fees. The authors would suffer from price increase the most, hence we are convinced that the authors, with the help of the editors, reviewers and journal’s Editorial Office should take proper care to bring their manuscripts into a shape corresponding to the journal’s style requirements*.

Another unfortunate editorial feature is the elimination of periods after abbreviations, the reason for which is not known. This is further compounded in *ZooKeys* in the citation of references where authors’ initials not only lack periods, but are written together and without a comma after the family name. Thus a 1974 paper by R. T. Abbott is listed as “Abbott RT (1974).” Perhaps it is this usage that is responsible for the disconnect in the authorship of *ZooKeys* papers by authors with Spanish surnames. As an example, the paper by Caballer has as authors “Manuel Caballer, Jesus Ortea, Samuel Narcisco.” The abstract of this paper posted on the *ZooKeys* web site shows authors as “Manuel Caballer Gutierrez, Jesus Ortea, Samuel Narciso.” If Caballer Gutierrez did not wish for his full name to be used, how did it get on the abstract? The abstracts of other papers by Spanish authors are similarly treated with the names on the abstract not in agreement with the names on the paper.

**Comment:** *It is a practice in many modern electronic journals to avoid periods after abbreviations and generally to simplify citation and reference style (see for example, reference style in PLOS and BioMed Central journals). Such a simplification makes the process of markup and text mining easier, which in turn facilitates the dissemination and use of the published information, to the benefit of the authors and science community as a whole.*

The list of *ZooKeys* Subject Editors for most groups is impressive, with some groups and areas being quite restricted. For Mollusca there is one editor for “terrestrial slugs, Northern Hemisphere” (Andrzej Wiktor) and another for “terrestrial gastropods, Northern Hemisphere” (Eike Neubert). The latter is in addition to a listing of Anatoly A. Schileyko as editor for “terrestrial gastropods.” The editor for Opisthobranchia is Nathalie Yonow. The only other Mollusca editor is Bruce A. Marshall for “shelled marine mollusks.” Other categories (Bivalvia, Scaphopoda, other mollusks) are listed without editors.

These editors are detailed here because there is a serious question as to why, with such specific appointments, molluscan *ZooKeys* submissions have apparently not been assigned to the appropriate editor. Of the seven papers published on shelled marine mollusks, the only one on opisthobranchs was edited by “Guest Editor Herman Strack”, presumably as it was written by the Opisthobranchia Editor Nathalie Yonow. Of the three papers on marine bivalves, one was edited by Marshall and two by Yonow. The only three papers on shelled marine gastropods were all edited by Neubert. An eighth marine mollusk paper, on cephalopods, was edited by Marshall. Dr. Marshall advises (personal communication 17 November 2012) that he has no knowledge of the papers on shelled marine mollusks that were edited by others.

**Comment:** *For some reasons, it was difficult to assemble an editorial group responsible for mollusks in ZooKeys. Probably this is due largely to the fact that the community studying this large group of animals has established specialized society journals, thus many active specialists on Mollusca are engaged elsewhere as editors*.

*Currently*, *ZooKeys is undertaking an initiative, called Global Editorial Networks (GENs)^2^ to extend the focus and scope of the journal to areas close to or beyond taxonomy in its narrow sense. Specialists who wish to serve as subject editors in various subject and taxa, Mollusca included, may apply using the following link^3^*.

Are authors in some way allowed to choose a *ZooKeys* editor? As shown above, neither the editors nor reviewers for this journal are expected to correct English usage. A cynical conclusion would be that *ZooKeys* is a venue for publishing a paper in which the English is less than minimal as an editor can be selected whose first language is not English — simply pay minimal page charges and bypass editing. I am not alleging that this has happened, but it is a possibility that is hard to ignore.

**Comment:** *Authors have no influence on selecting the editors of their manuscripts. Editors are assigned by the journal’s Editorial Office. We stress again that the language editing in ZooKeys is a responsibility of the authors. It would be quite an exaggeration to generalize that “Zookeys is a venue for publishing a paper in which English is less than minimal*” *based on a single or few examples of papers published in not properly edited English language*.

The presence of a number of American systematists on the *ZooKeys* Bioinformatics Advisory Panel argues against any prejudice toward American authors. On the other hand, not only are there are no American Editors for Mollusca, the only two whose first language is English are Marshall and Yunow (inclusion of the latter is a guess based on her background—it is suspected that her English is superior to mine). Why were the only three papers on shelled marine non-opisthobranch gastropods edited by an editor (Neubert) whose first language is presumably German? As Dr. Neubert has published many papers in fluent English the core problem appears to be the failure of the Author Guidelines to require any supervision or correction of English. Indeed, the wording of the Guidelines is such that reviewers and editors are expected to expend their efforts on scientific quality and style. However, there is a provision that an editor can reject a manuscript for “poor English.”

**Comment:** *There is no, nor has there ever been, prejudice against either American authors or editors, nor against such from any other nationality! Invitations to American malacologists to join the board of ZooKeys have been sent several times in the past, but unfortunately these have been declined*.

*The presence of many native English speakers in the Editorial team of ZooKeys is understandable and has a great value for the journal. On the other side, there are also many other excellent editors from non English-speaking countries, which reflects the truly international character of the journal and of taxonomy as a discipline in general*.

*It would be unrealistic to rely exclusively on the services of native English speakers as editors and reviewers, just because they are expected to correct the language of the submitted manuscripts*.

The *ZooKeys* web site, as of 17 November 2012, lists by category the 1,014 papers it has published. Of that number 891 are on Arthropoda. These numbers may not be current as 22 papers on Mollusca are listed but 26 have been counted.

**Comment:** *Numbers are derived from the articles’ metadata. The above figures will be checked and corrected*.

Now that *ZooKeys* has become an important publication venue in systematic zoology, it would be advantageous for the journalto use editors who are familiar with the subject matter. There is also an obvious need to find some way to insure that English is used in a manner that can be understood. Minor grammatical errors can be accepted but wholesale misuse of the language should not be allowed. Unless the editing process is tightened up, it is unlikely that the malacological community will make much use of the journal.

*ZooKeys* performed no favor for Simone by publishing his poorly presented paper. Accepting and publishing a work that is basically unintelligible was a disservice to Simone and to *ZooKeys* and detracts from the credibility of both.

**Comment:** *We fully agree with this general conclusion and thank you once again for raising the question. We are convinced that this case will be used to improve the control over the quality of English language editing of the manuscripts submitted to ZooKeys*.

## Footnotes

^1^ https://uspdigital.usp.br/tycho/CurriculoLattesMostrar?codpub=67A2A1A0A5C6

^2^ http://www.pensoft.net/journals/zookeys/about/Editorial%20Team#Global%20Editorial%20Networks%20%28GENs%29

^3^ http://pensoft.net/gen_form.htm
